# Cigarette Smoking Accelerated Brain Aging and Induced Pre-Alzheimer-Like Neuropathology in Rats

**DOI:** 10.1371/journal.pone.0036752

**Published:** 2012-05-11

**Authors:** Yuen-Shan Ho, Xifei Yang, Sze-Chun Yeung, Kin Chiu, Chi-Fai Lau, Andrea Wing-Ting Tsang, Judith Choi-Wo Mak, Raymond Chuen-Chung Chang

**Affiliations:** 1 Laboratory of Neurodegenerative Diseases, Department of Anatomy, The University of Hong Kong, Hong Kong SAR, China; 2 Research Centre of Heart, Brain, Hormone and Healthy Aging, The University of Hong Kong, Hong Kong SAR, China; 3 Department of Medicine, LKS Faculty of Medicine, The University of Hong Kong, Hong Kong SAR, China; 4 Department of Pharmacology and Pharmacy, LKS Faculty of Medicine, The University of Hong Kong, Hong Kong SAR, China; 5 State Key Laboratory of Brain and Cognitive Sciences, The University of Hong Kong, Hong Kong SAR, China; 6 Shenzhen Centre of Disease Control and Prevention, Shenzhen, China; Nathan Kline Institute and New York University School of Medicine, United States of America

## Abstract

Cigarette smoking has been proposed as a major risk factor for aging-related pathological changes and Alzheimer's disease (AD). To date, little is known for how smoking can predispose our brains to dementia or cognitive impairment. This study aimed to investigate the cigarette smoke-induced pathological changes in brains. Male Sprague-Dawley (SD) rats were exposed to either sham air or 4% cigarette smoke 1 hour per day for 8 weeks in a ventilated smoking chamber to mimic the situation of chronic passive smoking. We found that the levels of oxidative stress were significantly increased in the hippocampus of the smoking group. Smoking also affected the synapse through reducing the expression of pre-synaptic proteins including synaptophysin and synapsin-1, while there were no changes in the expression of postsynaptic protein PSD95. Decreased levels of acetylated-tubulin and increased levels of phosphorylated-tau at 231, 205 and 404 epitopes were also observed in the hippocampus of the smoking rats. These results suggested that axonal transport machinery might be impaired, and the stability of cytoskeleton might be affected by smoking. Moreover, smoking affected amyloid precursor protein (APP) processing by increasing the production of sAPPβ and accumulation of β–amyloid peptide in the CA3 and dentate gyrus region. In summary, our data suggested that chronic cigarette smoking could induce synaptic changes and other neuropathological alterations. These changes might serve as evidence of early phases of neurodegeneration and may explain why smoking can predispose brains to AD and dementia.

## Introduction

Epidemiological studies have shown that cigarette smoking is an important risk factor of cognitive decline and AD, the most common form of dementia [1]–[3]. Cigarette smoking not just doubles the risk of developing dementia and AD [4]; it also accelerates the rate of cognitive decline in elderly without dementia [2]. Apart from active smoking, recent study shows that exposure to secondhand smoke i.e. passive smoking can also increase the odds of developing cognitive impairment [5]. Subjects who have been exposed to secondhand smoke for more than 25 years and have history of carotid artery stenosis have a 3-fold increased risk for dementia [6]. Although these studies suggest a linkage between cigarette smoking (both active and passive) and cognitive impairment, there is insufficient experimental data demonstrating how smoking induces cognition-related pathological changes. In fact, early studies on human autopsy samples demonstrated conflicting results for the association between smoking and AD-neuropathological changes. For example, Sabbagh and colleagues had studied the association between smoking and AD in never, former and active smokers followed to AD autopsy. They found that smoking had no significant influence on AD neuropathology (Braak stage, neurofibrillary tangles, total plaques and neuritic plaques) regardless of *AP0E* ζ4 status, although higher levels of smoking were associated with shorter disease duration [7]. However, in another study conducted by Ulrich and colleagues, it was found that the amount of smoking was positively correlated with the neurofibrillary changes as expressed in Braak stages in smokers [8]. Tyas and colleagues also reported that former smokers had more neuritic plaques in the neocortex and the hippocampus than never smokers [9]. Although the presence of senile plaques and neurofibrillary tangles has been widely accepted as neuropathological hallmarks of AD, it is worth to notice that the number of senile plaques is not associated with duration and severity of dementia [10], [11]. On the other hand, synaptic pathology was closely associated to the clinical dementia in AD [10], [12]. Therefore other indicators apart from senile plaques and neurofibrillary tangles may be included to better study the relationship between smoking and neuropathological changes in AD.

For a long time, cigarette smoking has been known as an important environmental aging accelerator [13], [14] partly because it induces oxidative stress in multiple organs including the brain [15]. During the combustion of a cigarette, more than 4000 chemicals are produced, in which many of them are reactive radicals. These reactive radicals modify biomolecules through oxidation reaction, resulting in defective cellular signaling and accumulation of malfunctioned proteins [16]. A number of reports have shown that oxidative stress was found in the brains of cigarette smoke exposed-animals [17], [18]. However, oxidative stress can be generally found in many diseases, thus more cognition-related or neurodegeneration-related pathological changes should be presented to demonstrate a direct linkage between smoking and AD.

In this study, we adopted a passive smoking model in which rats were exposed to sham air or cigarette smoke 1 h daily for 8 weeks. We investigated if exposure to cigarette smoke could induce oxidative stress and early pathological changes that are related to cognitive impairment or development of AD. Since synaptic degeneration is an early event during normal aging and AD [19]–[21], we detected changes in synapse by measuring the levels of synaptic proteins synapsin-1, synaptophysin and PSD95. Stability of the spine and functions of axonal transport were elevated by measuring the expression of drebrin and acetylated-tubulin. The early pathological changes of AD were evaluated by measuring the levels of beta-amyloid (Aβ) peptide and hyperphosphorylation of the tau protein. Our data suggested that exposure to cigarette smoke could induce oxidative stress, changes in synaptic proteins and pre-AD-like pathological changes which might partly explain why passive cigarette smoking is harmful to the brain.

## Materials and Methods

### Antibodies and chemicals

Mouse monoclonal antibody against 8 hydroxyguanosine (8-OHG) (recognizes 8-hydroxy-2′-deoxyguanosine, 8-hydroxyguanine and 8-hydroxyguanosine) was from Abcam (Cambridge, UK). Rabbit polyclonal antibodies for tau pT231 against tau phosphorylated at Thr231, tau pT205 against tau phosphorylated at Thr205, tau pS396 against tau phosphorylated at Ser396 and tau pS404 against tau phosphorylated at Ser 404 were purchased from BioSource International (Hopkinton, MA, USA). Rabbit polyclonal antibody against amyloid precursor protein (APP) was from Novus Biologicals (Littleton, CO). Rabbit polyclonal antibodies against phosphorylated ERK (P44/42), total ERK, phosphorylated JNK (Thr183 or Tyr185), total JNK, cleaved caspase-3, phosphorylated GSK-3β (Ser9) and mouse monoclonal antibody against synapsin-1 were from Cell Signaling Technology (Boston, MA, USA). Rabbit polyclonal antibody against PSD-95 was from Synaptic Systems (Göttingen, Germany). Mouse monoclonal antibodies against acetylated-tubulin and α-tubulin were from Sigma-Aldrich, Inc. (St. Louis, MO, USA). Mouse monoclonal antibody against synaptophysin and rabbit polyclonal antibody against rodent Aβ were from Chemicon (Temecula, CA). Rabbit polyclonal antibody against human sAPPβ and mouse polyclonal antibody against human sAPPα were from IBL (Gunma, Japan). Secondary antibodies were horseradish peroxidase-conjugated goat anti-rabbit and goat anti-mouse from DAKO (Glostrup, Denmark). Alexa Fluor®-488 goat anti-rabbit IgG was purchased from Invitrogen (Carlsbad, CA). The protein content assay kit and Polyvinylidene fluoride (PVDF) membrane were purchased from Bio-Rad (Hercules, CA, USA). Enhanced chemiluminescence (ECL) detection kit was from Amersham (Buckinghamshire, UK). Rabbit polyclonal antibody against human-tau (K9JA/ pan-tau) and anti-fade mounting medium were from DAKO (USA). Mouse polycolonal antibody against GSK3β phosphorylated at Tyr 216 was from BD Biosciences (San Jose, CA, USA). Rabbit polyclonal antibody against PP2A phosphorylated at Tyr307 was from Epitomics (CA, USA).

### Animals

Nine male Sprague-Dawley rats (150–200 g) were purchased from the Laboratory Animal Unit (LAU) of the LKS Faculty of Medicine in the University of Hong Kong. The rats were maintained in a temperature-controlled room with a 12-h light/dark cycle throughout the observation period. The rats were divided into two groups, with four rats in the control group, five rats in the cigarette smoke (smoking) group. The control group were exposed to sham air, while the smoking were exposed to 4% cigarette smoke for 1 h daily with commercially available cigarettes (Camel; filter, R.J. Reynolds, Winston-Salem, NC, USA) for 56 days using the modified ventilated smoking exposure chambers as previously described by Chow *et al* [22], [23]. In brief, the rats of the smoking group were housed in a ventilated 20-litre chamber filled with smoke. The concentration of smoke was kept constant at 4% (vol/vol, smoke/air) by using a peristaltic pump (Masterflex; Cole-Parmer Instrument Co., Niles, IL, USA) to deliver fresh cigarette smoke from burning cigarettes at a constant rate (40 ml/min), while another pump was simultaneously used to deliver fresh air from outside at a constant rate (960 ml/min) to mix. Rats were exposed to 12 cigarettes in an hour daily. For the control group, the rats were subjected to the same procedures in another ventilated chamber but exposed only to fresh air (0%, vol/vol, smoke/air) simultaneously. This cigarette smoking model was proven to neither disturb the normal physiological functions of the animals, such as acid/base balance and O_2_/CO_2_ in the blood, heart rate and blood pressure, nor to impose any stress on the animals. The body weight was similar between the control and smoking groups [23]. After 56-day exposure, the rats were euthanized by overdose of pentobarbitone. Their brains were dissected for Western blot analysis and immunohistochemical analysis. This protocol was approved by the Committee on the Use of Live Animals in Teaching and Research (CULATR) of The University of Hong Kong.

### Immunohistochemical staining

Brain tissues were fixed in 4% paraformaldehyde for three days and dehydrated in ethanol and embedded in paraffin. 6-μm-thick coronal rat brain sections were cut. After dewaxing and rehydration, the sections were treated with 0.01 M citrate buffer (pH 6.0) with 0.1% Tween-20 at 90°C for 15 min for antigen retrieval. For sections stained with Aβ peptide, the sections were treated with 88% formic acid for eight minutes after citric buffer treatment. Sections were then washed in PBS three times then blocked with 10% normal goat serum in PBS for 1 h. Sections were incubated with primary antibody against 8-OHG (1∶100), synapsin-1 (1∶400), acetylated-tubulin (1∶800) or Aβ peptide (1∶100) at 4°C overnight. After washing with PBS, the sections were stained with secondary antibody, Alexa Fluor®-488 for 2 h and finally stained with DAPI to reveal the nuclei. Sections were then mounted with anti-fade mounting medium. The sections were examined with a Zeiss LSM510 confocal microscopic system (Carl Zeiss, Jena GmbH, Jena, Germany).

### Western blotting analysis

Total lysate was collected from rat hippocampus. In brief, tissue samples were homogenized in ice-cold lysis buffer (10 mM Tris-HCl (pH 7.4), 1 mM NaCl, 20 mM Na_4_P_2_O_7_, 2 mM Na_3_VO_4_, 1% Triton-X-100, 10% glycerol, 0.5% deoxycholate, 0.1% SDS, 1 mM phenylmethylsulfonyl fluoride, protease inhibitor cocktail and phosphatase inhibitor cocktail) and centrifuged, subsequently, the supernatant was collected. The samples were subjected to SDS 10% or 12.5% polyacrylamide gels electrophoresis and transferred to PVDF membranes. Non-specific binding on the membranes was blocked by 5% w/v non-fat milk in TBST (TBS containing 0.1% Tween-20) for 1 h to prevent non-specific binding. Primary antibodies were diluted in TBST and incubated with the membrane as follows: tau pT231 (1∶4000), tau pS396 (1∶4000), tau pS205 (1∶2000), tau pS404 (1∶3,000), pan-tau (1∶4000), phosphorylated ERK (1∶2000), total ERK (1∶4000), phosphorylated JNK (1∶2000), total JNK (1∶4000), synapsin-1 (1∶40,000), synaptophysin (1∶40,000), PSD95 (1∶2000), APP (1∶5000), sAPPα (1∶500), sAPPβ (1∶500), acetylated-tubulin (1∶40,000). After washing, the membranes were incubated with horseradish peroxidase-conjugated secondary antibodies for 1 h and subsequently developed by using the ECL or ECL-plus Western blotting detection kit. The membranes were then stripped with stripping buffer (50 mM glycine, 2% SDS, pH 2.0) and re-probed with anti-α-tubulin antibody (1∶40,000) and goat anti-mouse HRP secondary antibody. Optical density of the blots was measured with Image J software (National Institutes of Health, USA). The band intensities were normalized against α-tubulin and results were expressed as fold of control.

### Statistical analysis

Data were expressed as mean ± standard error (SE). The significance of the differences among different groups was determined by unpaired *t*-test. All statistical analyses were performed using SigmaStat® (Jandel Scientific, CA, USA). *P*<0.05 was considered to be statistically significant.

## Results

### Passive cigarette smoking induced oxidative stress in the rat hippocampus

**Figure 1:**
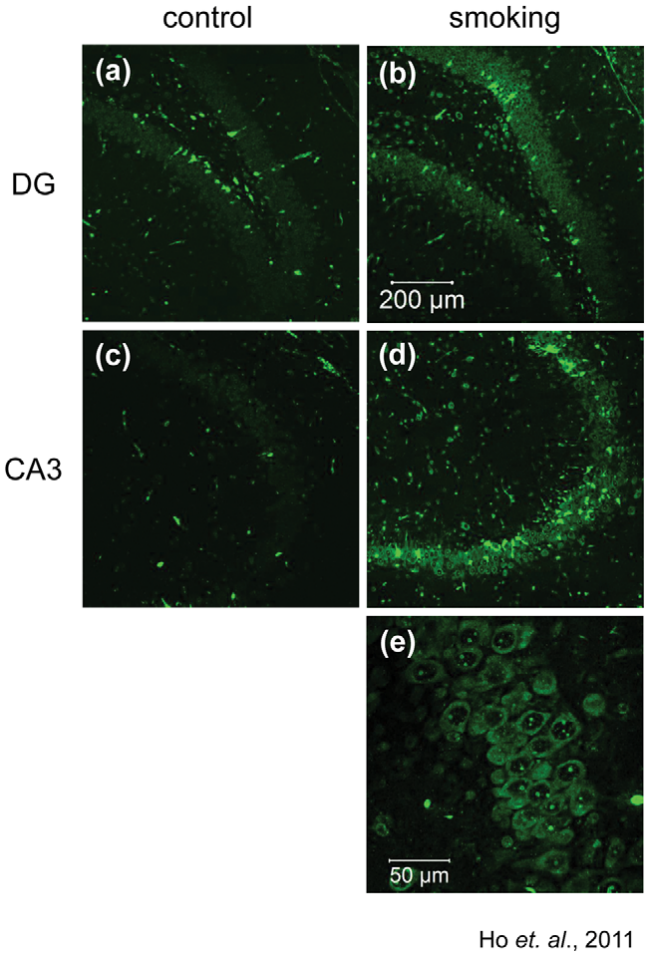
Level of 8-OHG was elevated in rat hippocampus exposed to cigarette smoking. Brain sections were stained with anti-8-OHG antibody to detect the level of oxidative stress. Representative photos were selected from the control group (a and c) and the smoking group (b and d), magnification, 100X. An enlarged photo of the CA3 region of the smoking group was shown in (e), magnification, 400X.

It has been found that passive cigarette smoking can induce oxidative stress in both the peripheral circulation and in the central nervous system (CNS) [17], [24]. The first part of this study was to confirm the presence of oxidative stress in the hippocampus of the rats which had been exposed to cigarette smoke for 56 days. 8-OHG served as a marker for oxidative stress. We found that the immunoreactivity of 8-OHG was markedly increased in the dentate gyrus and the CA3 regions of the hippocampus of the smoking group (Fig. 1a to e), suggesting an elevation of oxidative stress in these regions.

### Passive cigarette smoking affects synaptic proteins in the hippocampus

**Figure 2:**
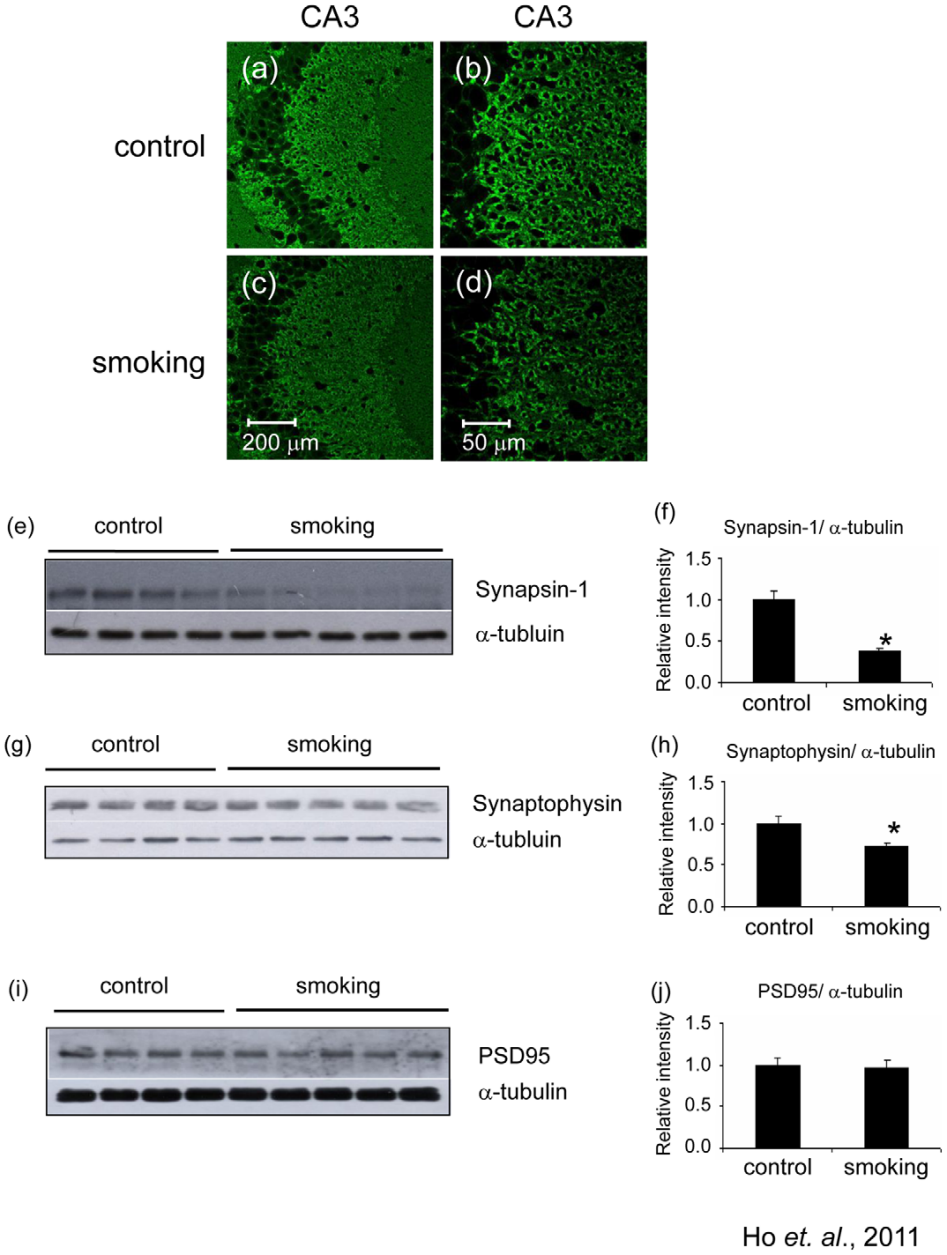
Levels of the pre-synaptic proteins were decreased in the hippocampus of the smoking group. Brain sections were stained with anti-synapsin-1 antibody. The CA3 region of (a) control group and (c) smoking group were shown, magnification, 200X; with corresponding enlarged images shown in (b and d), magnification, 400X. Western blot analysis was performed on the total lysate of the whole hippocampus, confirming the decrease of synapsin-1 in the hippocampus of the smoking group (e); quantitative analysis of the band intensity of synapsin-1 was shown in (f). Western blot analysis showed that the levels of synaptophysin in the hippocampus were decreased in the smoking group (f); quantitative analysis of the band intensity of synaptophysin was shown in (g). Western blot analysis showed that the levels of PSD95 in the hippocampus were similar among the two groups (h); quantitative analysis of the band intensity of PSD95 was shown in (i). *P<0.05 compared to control.

**Figure 3:**
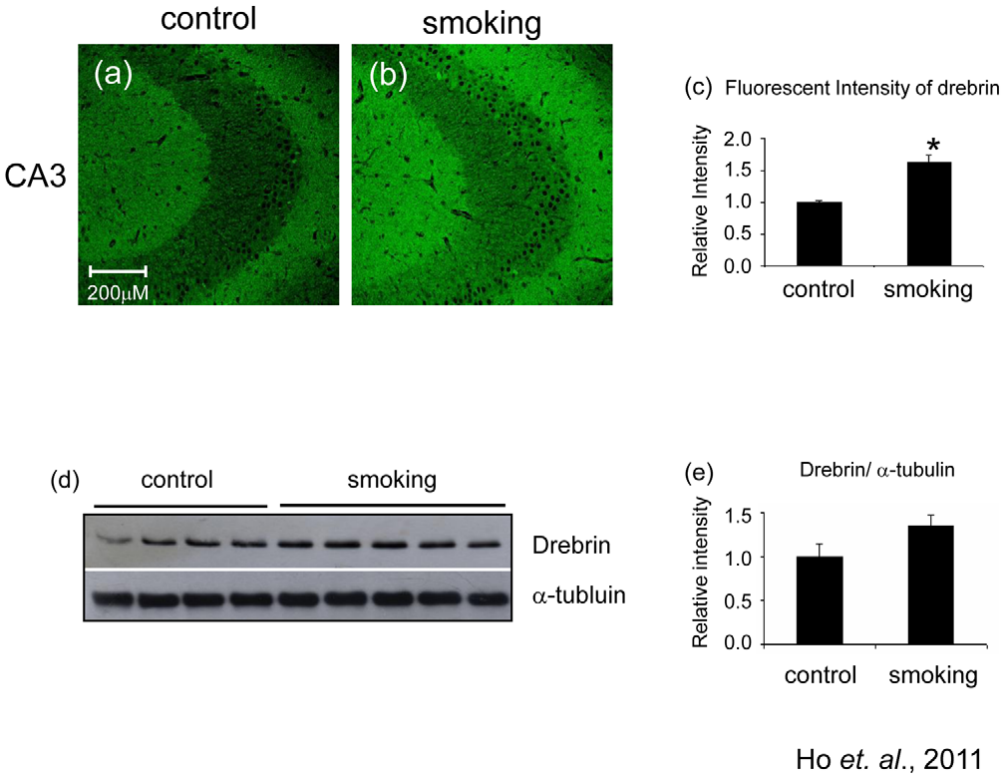
Level of drebrin was increased in the smoking group. Brain sections were stained with anti-drebrin antibody. The CA3 region of the control (a) and smoking (b) group were shown, magnification, 100X. Western blot analysis showed that there was a trend of increase in the band intensity of drebrin but it is not statistically significant (c and d).

Synaptic proteins are important for the normal functioning of synapses. To investigate the effects of smoking on synapses, we investigated the expression of some pre-synaptic and post-synaptic proteins. Immunohistochemical staining revealed that the level of pre-synaptic protein synapsin-1 was markedly reduced as demonstrated by the reduction of fluorescent intensity (Fig. 2a to d). The result was confirmed by Western blot analysis (Fig. 2e and f) which showed that the level of synapsin-1 decreased to 0.38±0.03 fold of control in the smoking group. We also detected the level of synaptophysin (Fig. 2g), which is another pre-synaptic protein. The band intensity of synaptophysin decreased to 0.73±0.03 fold of control in the smoking group (Fig. 2h). To investigate the effects of smoking on the post-synaptic region, we detected the level of PSD95. We found that there was no significant difference of the levels of PSD95 between the control and smoking group (Fig. 2i and j). To investigate the impact of smoking on spine plasticity and stability, we performed immunohistochemical staining on drebrin, which is found extensively in the dendritic spine responsible for receiving excitatory inputs. The level of drebrin in the hippocampus was markedly elevated (Fig. 3a and b). The fluorescent intensity in the smoking group increased to 1.62±0.11 fold of control (Fig. 3c). Western blot analysis showed a trend of increase in band intensity, although it was not statistically significant (Fig. 3d and e). Our data suggested that smoking could have impact on both the pre-synaptic and the post-synaptic regions.

### The level of acetylated-tubulin was decreased in the smoking group

**Figure 4:**
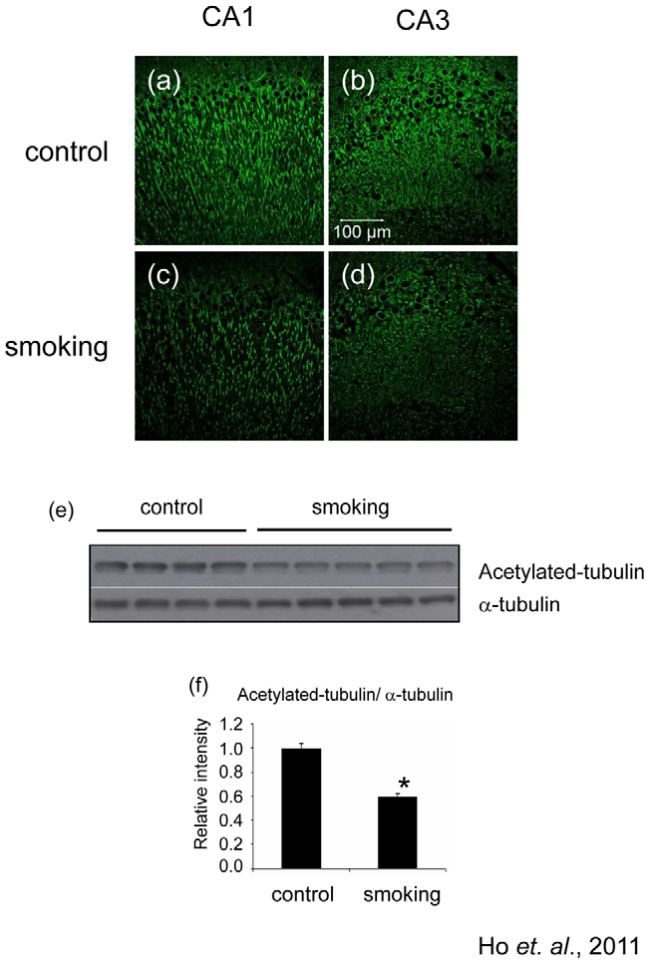
Level of acetylated-tubulin was decreased in the hippocampus of the smoking group. Brain sections were stained with anti-acetylated-tubulin antibody. The CA1 and CA3 regions of control group (a and b), smoking group (c and d) were shown, magnification, 200X. Western blot analysis confirmed the decrease of acetylated-tubulin in the hippocampus of the smoking group (e); quantitative analysis of the band intensity was shown in (f). *P<0.05 compared to control.

Since smoking may also affect other cellular functions such as the motor-based trafficking system, we detected the levels of acetylated α-tubulin which can affect the binding affinity of kinesin-1. The immunoreactivity of acetylated-tubulin was markedly decreased in the CA1 and CA3 regions of the hippocampus in the smoking group (Fig. 4a to d). Quantitative analysis of the Western blot results suggested that the level of acetylated-tubulin in the smoking group was just 0.59±0.03 fold of the control group (Fig. 4e and f). This alteration of acetylation levels of α-tubulin might imply an impairment of cellular transport system.

### Passive cigarette smoking induced phosphorylation of tau in the hippocampus

**Figure 5:**
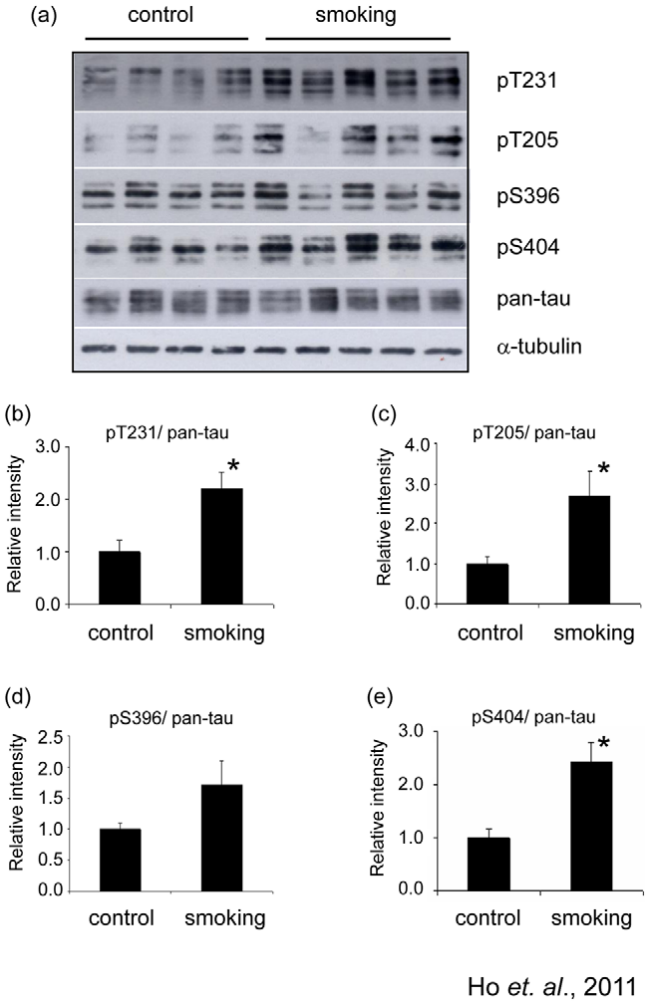
Smoking induced phosphorylation of tau. The total lysate of the hippocampus of the rats were subjected to Western blotting analysis. Phosphorylation of tau was detected by pT231 (reacts with phosphorylated tau at Thr 231), pT205 (reacts with phosphorylated tau at Thr 205), pS396 (reacts with phosphorylated tau at Ser 396) and pS404 (reacts with phosphorylated tau at Ser 404). Total tau was detected with the antibody pan-tau. α-tubulin was used as loading control. *P<0.05 compared to control.

The change in levels of acetylated-tubulin made us suspect that smoking might have other effects on the cellular transport machinery. The level of phosphorylation can affect the binding affinity of tau to microtubules (MTs), hence the stability of the cytoskeleton. We examined some phosphorylation sites of tau in the hippocampus of our rats. Western blots developed with pan-tau (K9JA) antibody indicated that the total tau level was not altered by smoking (Fig. 5a). Phosphorylation of tau was detected by Western blots developed with phosphorylation-dependent and site-specific tau antibodies. Among the four phosphorylation sites we studied, smoking induced a significant increase in tau phosphorylation at Tyr231, Tyr 205 and Ser 404 (Fig. 5a), where the changes were 2.19±0.03, 2.70±0.60 and 2.44±0.36 fold of control, respectively (Fig. 5b, c, e). There was no significant difference for the levels of phosphorylated tau at Ser396 between the control and smoking groups (Fig. 5d).

### APP processing was altered by passive cigarette smoking

**Figure 6:**
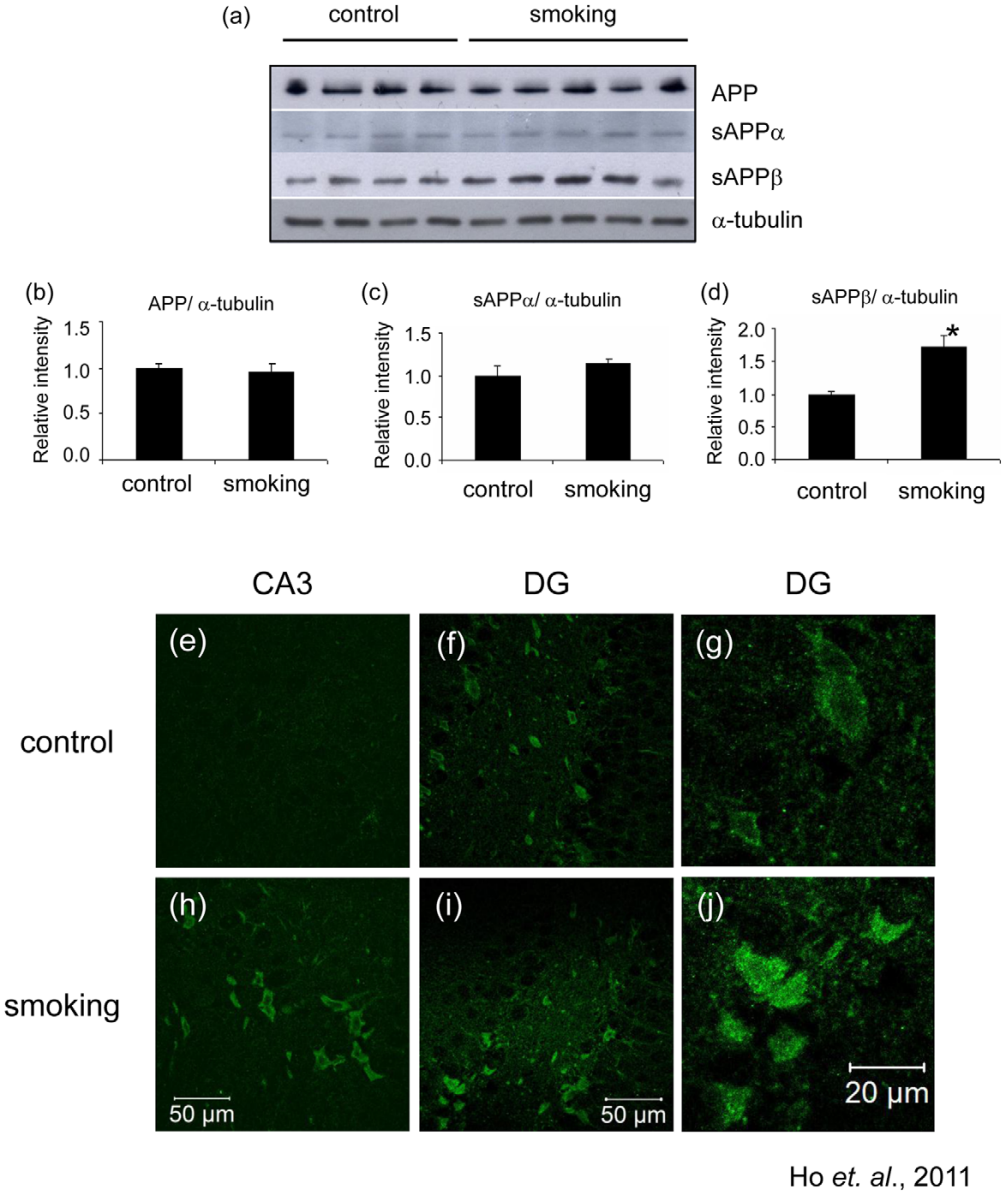
Smoking induced alteration of APP processing. The total lysate of the hippocampus of the rats were subjected to Western blotting analysis. The levels of APP, sAPPα and sAPPβ were detected (a); quantitative analysis of the band intensity was shown (b to d). *P<0.05 compared to control. Brain sections were stained with anti-rodent Aβ antibody. Photos of the CA1 and dentate gyrus were presented. Positive staining were increased in the smoking group (h and i) when compared to the control group (e and f), magnification, 400X. Enlarged photos of the dentate gyrus of the control (g) and smoking group (j) were shown. Note that Aβ was accumulated in the cell body (j).

Increased phosphorylation of tau and synaptic changes can be found in AD. Hence, we further detected AD-related pathological changes in our rats. We first detected the levels of APP but we found that there was no change in the expression of this protein (Fig. 6a and b). During the progression of AD, the enzymatic cleavage of APP by α-secretase and β-secretase is altered which results in increased productions of sAPPβ and in turn, Aβ peptide. We found that rats exposed to cigarette smoke had an increased level of sAPPβin their hippocampus, while the level of sAPPαwas unchanged compared to the control group (Fig. 6a, c and d). To confirm if there was an increased production of Aβ, we performed immunohistochemical staining on brain sections using the anti-rodent-Aβ antibody. The level of Aβ was markedly increased in the smoking group, particularly in the CA3 region. In the control animals, there was very little staining of Aβ in CA3 (Fig. 6e), but Aβ accumulated in the cell body in the smoking group (Fig. 6h). In the dentate gyrus, the basal level of Aβ in the control group (Fig. 6f and g) was higher than that in the CA3, but even then we could still detect an elevation in the staining of Aβ in the smoking group (Fig. 6i and j). Our results suggested that smoking could alter APP processing and direct it to the amyloidogenic pathway to favor the generation of Aβ peptide.

### Stress kinases were activated by passive cigarette smoking

**Figure 7:**
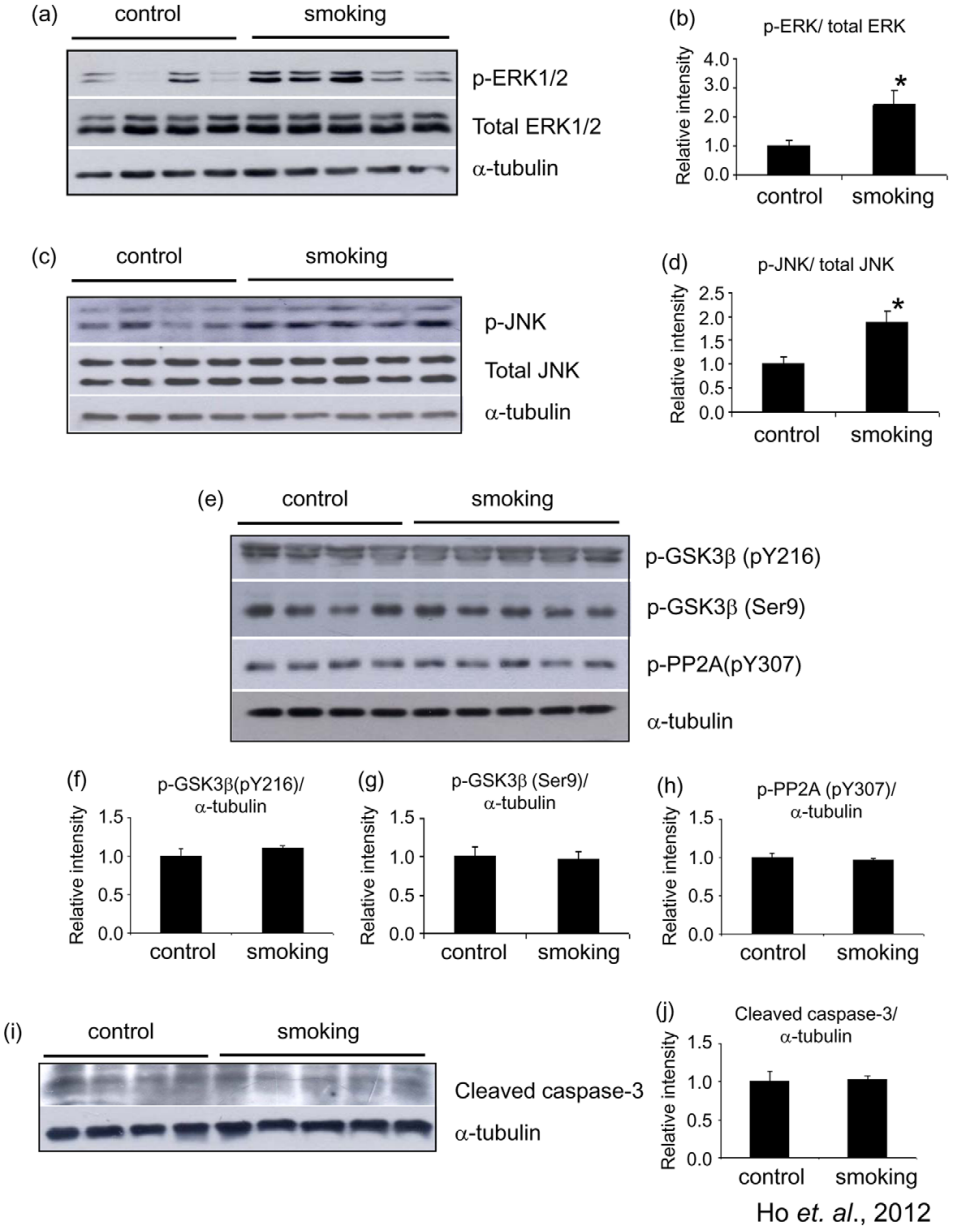
Smoking induced activation of stress kinases. The total lysate of the hippocampus of the rats were subjected to Western blotting analysis. The levels of p-ERK1/2, total ERK1/2 (a), p-JNK and total JNK (b), p-GSK3β (Ser9), p-GSK3β (pY216), p-PP2A (pY307) (e) and cleaved caspase-3 (i) were detected. α-Tubulin was used as loading control. Quantitative analysis of the band intensity of the detected kinases was shown in (b, d, f–g, and j). *P<0.05 compared to control.

Since passive cigarette smoking induced oxidative stress in our model, we further detected the possible activation of some stress kinases. Western blot analysis revealed that the level of p-ERK1/2 was increased to 2.42±0.48 fold of control, while the level of total ERK was unchanged (Fig. 7a and b). The level of p-JNK was also increased. The band intensity of the smoking group was increased to 1.87±0.23 fold of control (Fig. 7c and d). The levels of p-GSK3β (Ser9) (which represents the inactivation of kinase activity), p-GSK3β (pY216) (which represents the activation of kinase activity) and p-PP2A (pY307) were found to be similar among the control and the smoking groups (Fig. 7e to h). There was also no change in the levels of cleaved caspase-3 (Fig. 7i and j).

## Discussion

Cigarette smoking is a well-reported aging accelerator. The impact of smoking is reflected on both the reduction of lifespan and the increased susceptibility to diseases such as cardiovascular diseases, cancers and respiratory complications. Both active and passive smoking are associated with cognitive decline and AD [1], [3]–[6], yet the biological mechanism is not well characterized. In this study, we examined the pathological changes in the hippocampus of cigarette smoke-exposed rats. This model aims to imitate the situation of humans in restaurants or bars where cigarette smoking is permitted [22]. Our data suggested that daily exposure to cigarette smoke could accelerate the aging of the brain through inducing changes of synaptic proteins and pre-AD-like neuropathology.

The induction of oxidative stress is a major mechanism for cigarette smoke to impose its deleterious effects. It has been found that smokers have lower serum levels of antioxidants (ascorbic acid, α-carotene, β-carotene, cryptoxanthin, melatonin), anti-oxidative enzymes (selenium glutathione peroxidase, glutathione reductase) and higher levels of oxidative stress markers (malondialdehyde) [25]–[27]. This systemic oxidative stress explains why cigarette smoke has effects on multiple organs. Previous studies on human subjects confirm the increased free radical damage in the cerebral cortex in both smoker and AD patients [28]. A recent study also demonstrated the importance of oxidative stress by showing that Vitamin E can attenuate cigarette-smoke induced elevation of acetylcholinesterase activity and lipid peroxidation level in rat brains [29]. Therefore, we first characterized our model to confirm the presence of oxidative stress in the hippocampus of cigarette smoke-exposed rats. We used an antibody which could stain for 8-hydroxy-2′-deoxyguanosine (8-OHdG) and 8-hydroxyguanosine (8-OHG) to indicate the presence of oxidative stress. 8-OHdG and 8-OHG are generated when the guanine of DNA and RNA are oxidized by reactive free radicals respectively [30]. Studies have shown that the levels of 8-OHG and 8-OhdG are increased in vulnerable neurons of patients with AD, furthermore, the concentration of 8-OHdG in the CSF of AD patients was positively correlated with the duration of illness [31]–[33]. Our data showed that the level of 8-OHG was increased in the smoking group. This data is consistent with a report showing elevation of oxidative stress in the hippocampus of 60-day cigarette smoke-exposed mice [17]. Although another study conducted by Fuller and colleagues showed no change in oxidative stress levels in the brain of cigarette smoke-exposed rats [34], we believe the experimental protocol which determined the amount of cigarette smoke being taken was responsible for the observed discrepant findings. In their study, rats were exposed to cigarette smoke 3 h daily for 3 weeks to mimic airborne exposure as experienced in a household room, with a smoker consuming two cigarettes per hour over 10 h. While apoptotic cell death and increased activities of capsase-3 were found in their model, our model did not induce activation of caspase-3 nor neuronal cell loss as revealed by Nissl staining (data not shown). Data from human autopsy samples showed that the degree of pathological change (grey matter and white matter volume) is correlated to the number of cigarettes consumed and smoking duration [35], [36]. Thus the difference in experimental protocol may account for the discrepancy in findings between the two studies. Nevertheless, oxidative stress was present in our model which is consistent with the findings in AD patients [31]–[33]. Although we did not study other oxidative stress markers in our brain samples, it was found that the activities of superoxide dismutase and catalase, which reflect an antioxidant defense mechanism, were significantly elevated in the lungs of the smoke-exposed rats [23]. This further supports the induction of systemic oxidative stress after cigarette smoke exposure.

Synaptic degeneration is an early event in neurodegenerative diseases. Reduction in the number of synapses has been reported in normal aging human subjects and AD patients [37] Synaptic proteins are essential components to maintain normal synaptic function. Synaptophysin is the most abundant synaptic vesicle protein and is often used as a marker for quantifying the number of intact synapses. Synaptophysin interacts with other synaptic proteins such as synaptobrevin to control the exocytosis of synaptic vesicle, hence the release of neurotransmitters [38]. Synapsin-1 is another presynaptic protein which regulates neurotransmitter release. Through changing its state of phosphorylation, synapsin-1 controls the fraction of synaptic vesicles available for release [39]. Our data showed that chronic exposure to cigarette smoke decreased the expression of synapsin-1 and synaptophysin, which are signs of synaptic degeneration. Furthermore, we observed an increase in the expression of drebrin, a protein which is localized at the dendritic spine. Actin filaments are the major cytoskeletal component in dendritic spine. Drebrin can bind to actin and inhibit its interaction with myosin, resulting in reduction of contractile force of actomyosin and thereby inhibit spine retraction. Overexpression of drebrin has been shown to alter spine shape [40], [41]. Since morphological changes of spines are highly correlated to synaptic plasticity, it is possible that the increased expression of drebrin would affect normal synaptic functions. Dysregulation of drebrin expression has been found in AD patients and subjects with mild cognitive impairment [42], [43]. In fact, a recent study has shown that the expression of drebrin was increased in aged-rats with cognitive impairment but not altered in aged-rats without cognitive impairment [44]. It has been proposed that increased expressions of drebrin may have inhibitory effects on activity-responsive reorganization of spine structure, leading to a maladaptive rigidity of synaptic structure that could affect synaptic plasticity [44]. Hence, our data on synaptophysin, synapsin-1 and drebrin suggest that chronic exposure to cigarette smoke leads to synaptic changes which are related to aging and cognitive impairment.

Tubulin is the basic component of the MTs. Acetylation of α-tubulin is important for axonal transport and acetylated-tubulin is found on stable MTs. Kinesin-1, a motor protein which supports cargo-transport, can bind to MTs only when α-tubulin is acetylated [45]. Reduction of acetylated-tubulin has been reported in neurofibrillary tangle bearing neurons of AD patients [46]. We found that the level of acetylated-tubulin in cigarette-smoke exposed group was about 40% less than that in the control group, suggesting a possible impairment of axonal transport. More functional studies may be required to confirm this issue. Apart from the possible alteration of cellular transport machinery, the reduction of acetylated-tubulin may also reflect a decreased level of stable or mature MTs. The tau protein is a MT-associated protein which plays major role in promoting assembly and stability of MTs and vesicle transport. Hyperphosphorylated tau proteins lack the affinity to MTs and they are prone to self-associate into paired-helical filament structure and aggregates to form neurofibrillary tangles [47]. In our study, chronic exposure to cigarette smoke induced hyperphosphorylation of tau at Thr 231, Thr 205 and Ser 404. These changes can affect the normal functions of tau, leading to reduced stability of microtubule as reflected by the reduction of acetylated-tubulin.

The phosphorylation state of tau is determined by tau kinases and phosphatase such as GSK3β, CDK5, ERK1/2, JNK and PP2A [48]. We found that the levels of p-ERK1/2 and p-JNK were elevated in the hippocampus of the smoking group. We also detected the levels of p-GSK3β (Ser9), p-GSK3β (Tyr216) and p-PP2A (pY307) and found that their levels remained unchanged. The kinase(s) responsible for hyperphosphorylation of tau in our model is unclear. We proposed that hyperphosphorylation of tau by passive cigarette smoking could be a consequence of oxidative stress-mediated JNK and ERK activation. It has been shown that oxidative stress can activate ERK and JNK [49], [50], and the activation of these kinases can phosphorylate tau at Thr 231, Thr 205 and Ser 404 [48], [51]. Many components of cigarette smoke are potent oxidant. Nicotine, for example, has been shown to induce reactive oxygen species in rat mesencephalic neurons [52]. Chronic administration of nicotine to transgenic AD mice can exacerbate tau pathology [53]. As the level of 8-OHG was increased in the smoking group in our model, the oxidative stress being produced can be one of the activator for JNK and ERK, which in turns phosphorylated tau.

Oxidative stress may participate in the pathogenesis of AD through modulating APP processing. *In vitro* studies have shown that oxidative stress can modulate the activities of β- and γ-secretases and promote the production of Aβ through a JNK-dependent pathway [54], [55]. In our study, we detected an increase in immunoreactivity of Aβ in brain sections of the smoking group. This staining was performed by using an antibody which reacts with all isoforms of rodent Aβ but not human Aβ. It is possible that our Aβ staining did not result from Aβ peptide only but also from APP. However, we have shown that the level of APP remains unchanged between the control and smoking groups. It is therefore unlikely that the elevated levels of Aβ immunoreactivity were due to increased expressions of APP. The level of sAPPα (produced by α-secretase) was unchanged and that of sAPPβ (produced by β-secretase) was increased. Whether cigarette smoke affects the expression of the α- and β-secretases or alters their activities deserves further studies. Aβ toxicity plays a central role in the development of AD. Aβ peptide can induce synaptic degeneration and impairment of axonal transport [56]. Recent papers show that Aβ decreases acetylation of α-tubulin [57], [58]. It is likely that the Aβ induced by cigarette smoke is partly responsible for the observed reduction of acetylated-tubulin and synaptic changes.

This study has demonstrated that chronic exposure to cigarette smoke induced changes in synaptic proteins, changes in the dynamic of cytoskeleton and pre-AD-like neuropathology. Because we did not study the cognitive functions in these rats, we are not able to tell if these neuronal changes are directly linked to cognitive impairment. However, it is worth to note that the changes we observed may only reflect early pathological changes in pre-symptomatic stage, so cognitive impairment might not necessary appear. Senile plaques and neurofibrillary tangles were also absent in our model (data not shown). After all, cigarette smoke is only a risk factor for AD, the development of the disease can be affected by other factors such as midlife hypertension, physical inactivity and genetic factors. Smoking alone is unlikely to induce AD. We have no intention to claim that exposure to cigarette smoke can result in AD. Epidemiological studies suggest that the second largest number of AD cases worldwide was potentially attributed to smoking [59]. With this evidence in mind, we aimed to find out some early changes in the brain after cigarette-smoke exposure. These changes, such as phosphorylation of tau in several epitopes, do not represent a harmful event to the neurons if they occur alone. However, many abnormalities were observed in the hippocampus in the smoking-exposed rats and these changes collectively may have an impact in normal cellular functions. To conclude, our study demonstrated that exposure to cigarette smoke could induce pathological changes in the brain, and these changes might make us more susceptible to the development of cognitive impairment or even AD in late life. Further study is conducting to confirm the importance of oxidative stress in the process.
